# Oxidized Cellulose (Surgicel) Causing Postoperative Cauda Equine Syndrome

**DOI:** 10.7759/cureus.1500

**Published:** 2017-07-21

**Authors:** Tarush Rustagi, Kunal Patel, Sujit Kadrekar, Akshay Jain

**Affiliations:** 1 Department of Spine Surgery, Indian Spinal Injuries Center, New Delhi; 2 Department of Orthopedics and Joint Replacement, Punit Orthopedic Hospital, Mumbai; 3 Department of Orthopedics, Lokmanya Hospital, Pune; 4 Department of Orthopedics, SAIMS, Indore

**Keywords:** oxidised cellulose, cauda equina syndrome, hemostasis, cauda equina, surgicel

## Abstract

Hemostatic agents are often used in spine surgery to control excessive bleeding. Oxidized cellulose (OC) is a common hemostatic agent used for this purpose. We present a case of postoperative cauda equina syndrome caused by Surgicel (Johnson & Johnson, New Jersey, US). An emergent decompression led to complete recovery. All attempts should be made to remove OC before closure after hemostasis has been achieved.

## Introduction

In the event of a hemorrhage, hemostasis naturally occurs by means of vasoconstriction, platelet aggregation, and coagulation factors. During surgery, however, it is not always possible to wait for the natural processes of hemostasis to work. As a result, additive methods to achieve a stable coagulum are necessary. A variety of hemostatic agents is currently available [1]. Oxidized cellulose (OC) is frequently used to stop bleeding in spine surgery and to pack cavities or lytic defects. OC causes necrosis and swells up to form a gelatinous matrix, hastening clot formation [1]. Presented here is a case of cauda equina syndrome secondary to the OC swelling used for packing a vertebral body lytic defect in the lumbar spine.

## Case presentation

**Figure 1:**
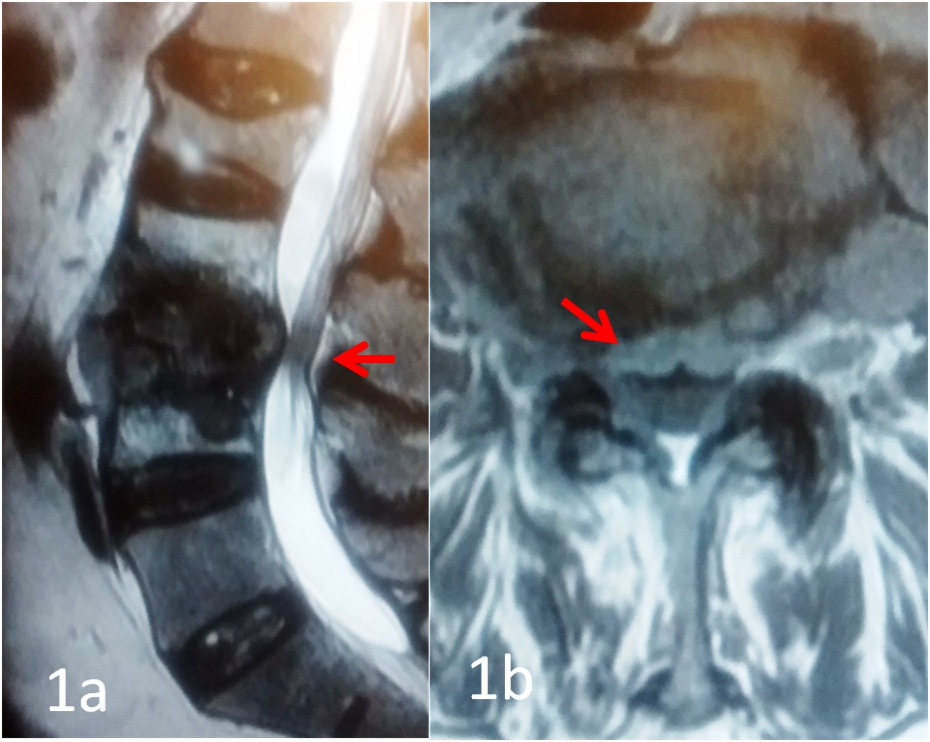
T2W sagittal and axial MRI scans showing L3-4 spondylodiscitis with compression of the thecal sac T2W sagittal and axial magnetic resonance imaging (MRI) scans showing L3-4 spondylodiscitis with compression of the thecal sac (red arrow)

A 52-year-old male presented in the emergency room with rapidly progressive weakness in both lower limbs along with significant back pain. At presentation, he had bilateral lower limb paresthesias. Motor strength was Medical Research Council (MRC) grade two: bilateral knee extension; grade three: right ankle dorsiflexion; and grade three: both extensor hallucis longus (EHL). The bowel bladder was not involved. A magnetic resonance imaging (MRI) scan showed L3-4 discitis with a significant compression of the thecal sac, as shown in Figures 1a and 1b.

**Figure 2:**
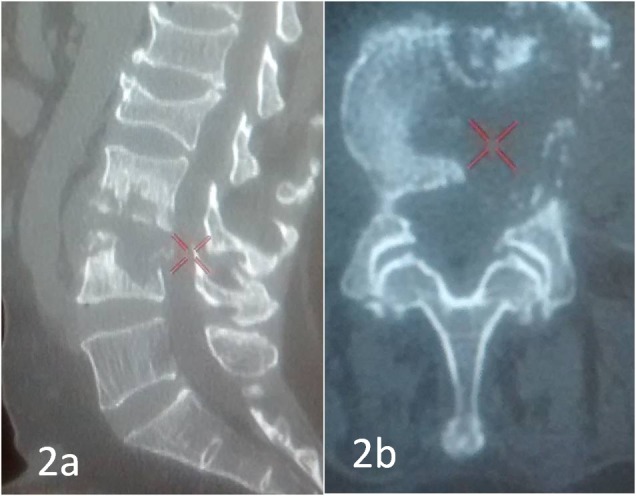
Sagittal and axial CT scans showing lytic destruction of the L3-4 vertebral bodies Sagittal and axial computed tomography (CT) scans showing lytic destruction of the L3-4 vertebral bodies (red X)

A computed tomography (CT) scan suggested the lytic destruction of the L3 and L4 bodies, as shown in Figures 2a and 2b.

The patient was ambulatory until a day prior to presentation. Considering the rapid course of neurological deterioration, an informed decision was made, in conjunction with the patient, for surgical intervention. A posterior decompression and stabilization from L1 to S1 was done. A biopsy was obtained and studied. Excessive bleeding was encountered from the L4 vertebral body defect. The lytic cavity was packed with Surgicel, which controlled the bleeding. After initial hemostasis was obtained, the OC was removed. This again led to significant bleeding and it was decided to leave the OC pack in the defect.

The patient showed an improvement in lower extremity motor strength and paresthesias at an eight-hour postoperative examination. 

**Figure 3:**
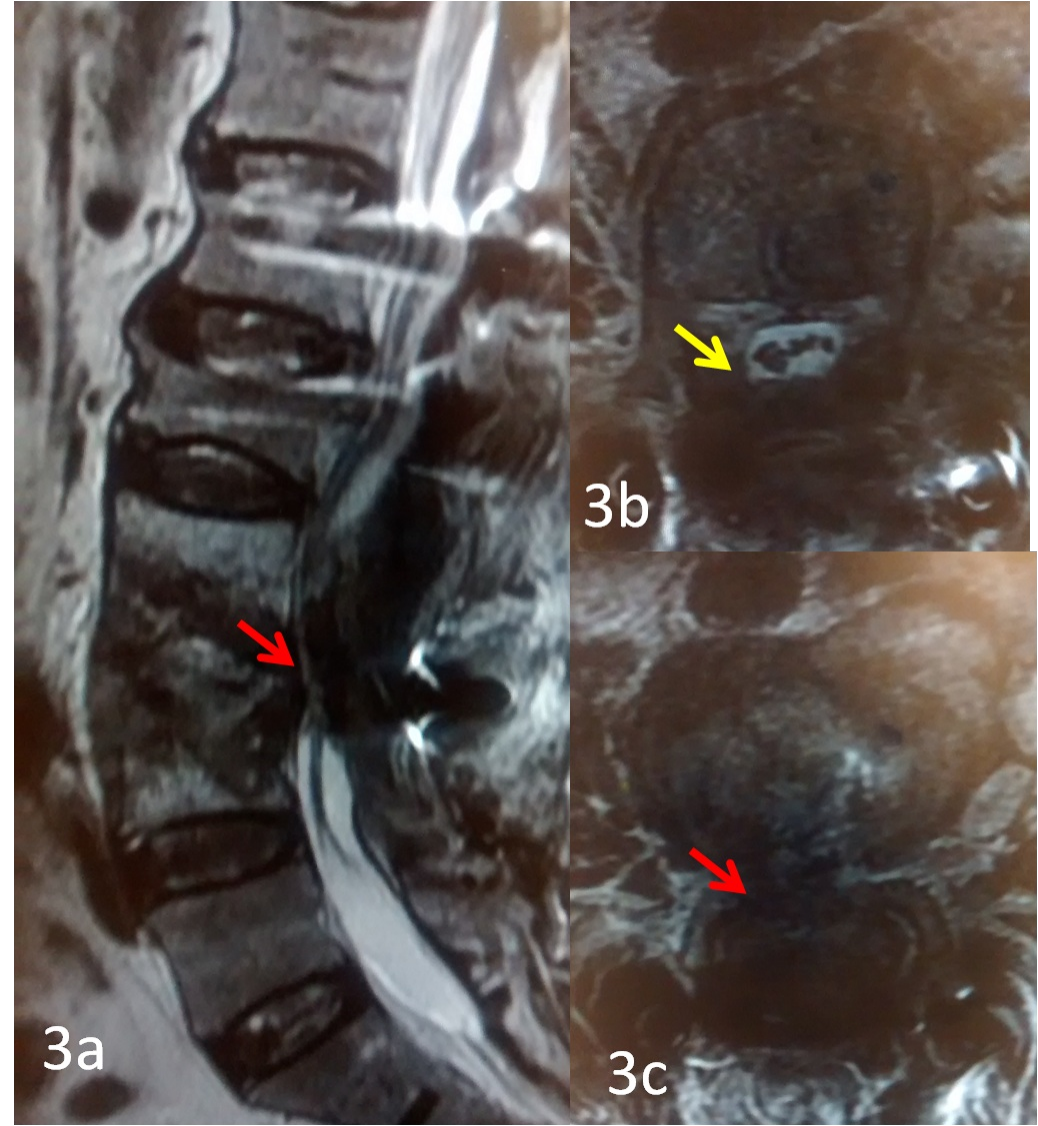
MRI scans of T2W sagittal and axial sections showing marked compression of the thecal sac at L3-4 level Magnetic resonance imaging (MRI) of T2W sagittal showing compression of the thecal sac (3a, red arrow). 3b is the axial image at L2-3, showing no thecal sac compression, for comparison (yellow arrow). 3c shows the axial image at L3-4 with the complete occlusion of the thecal sac (red arrow).

During evening rounds (12 hours after surgery), the patient again complained of numbness in both lower limbs and on examination, presented with grade-two power (MRC) in the bilateral ankle dorsiflexion and EHL along with reduced perianal sensation. An urgent MRI scan demonstrated the compression of the thecal sac at L3-4, as shown in Figures 3a, 3b, and 3c.

The finding was not suggestive of a hematoma collection. The site was re-explored on an urgent basis and a large coagulum of OC was found to be compressing the ventral aspect of the thecal sac. Upon removal, there was no residual bleeding from the vertebral body defect and the incision was closed in layers.

After re-exploration, the patient's neurological status improved. At two weeks post surgery, there was a complete recovery in the bilateral lower limbs.

**Figure 4:**
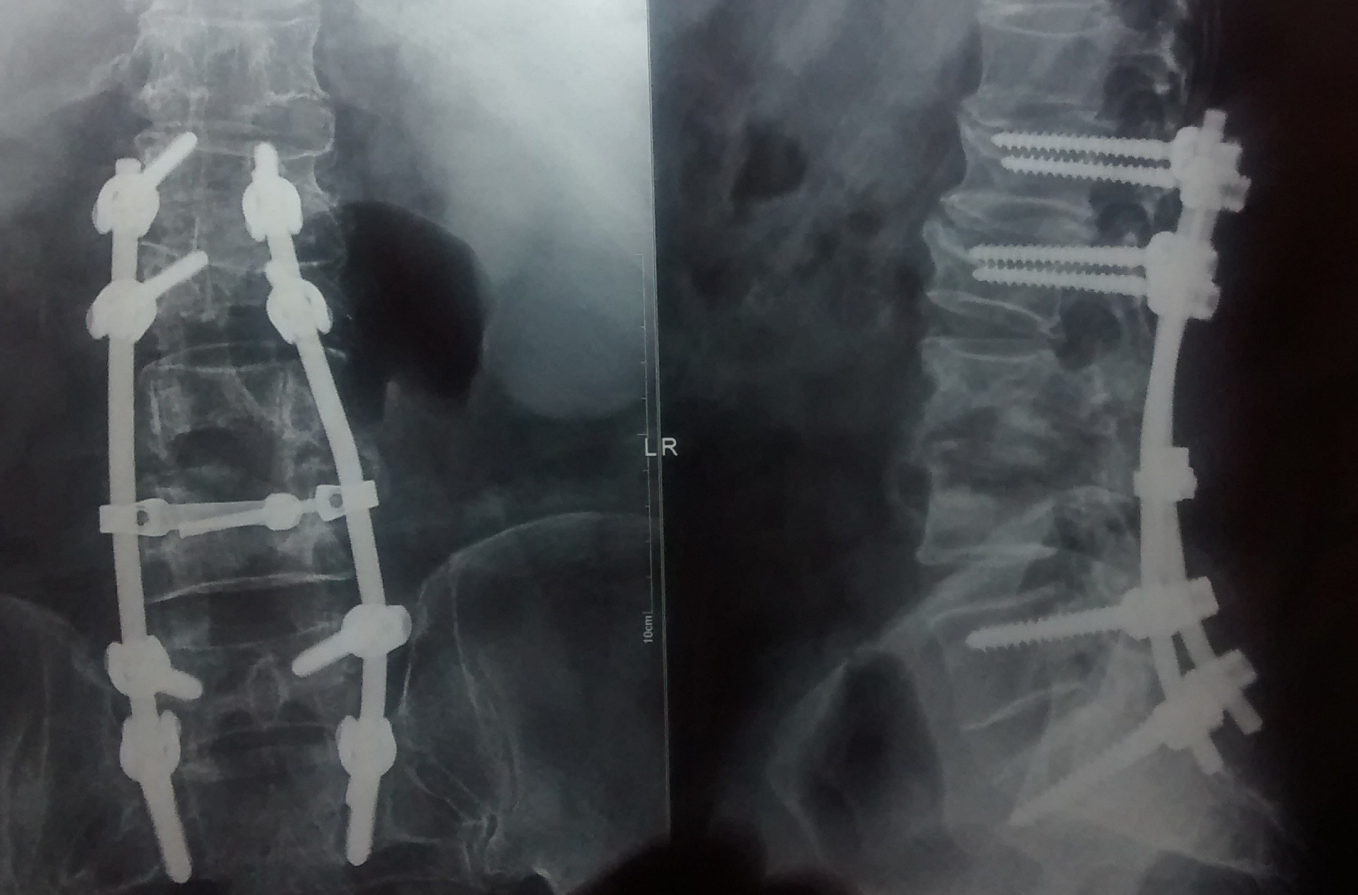
Postoperative X-rays at the two-year follow-up Postoperative X-rays at the two-years follow-up showing a stable construct

The biopsy confirmed tuberculosis. The patient was started on an antituberculosis regimen. At his last follow-up at two years, he is ambulatory with no complaints. His final X-rays of the lumbar spine show a stable construct, as shown in Figure 4.

## Discussion

In our case, we found that the coagulum of OC that was formed grew to a large volume, resulting in the ventral compression of the cauda equina. A surgical exploration and removal of the coagulum resulted in complete neurological recovery.

The methods of hemostasis may be broadly divided into thermal, mechanical, or chemical [2-3]. The thermal methods include the use of electrocautery while the mechanical methods include the use of pressure or ligatures and have been used traditionally. The chemical method, on the other hand, involves the use of chemicals to bring about hemostasis by inducing clot formation.

Various local agents are commonly available for use in spine surgery and are listed in Table 1.

**Table 1:** Common hemostatic agents in spine surgery

Table 1: Common Hemostatic Agents in Spine Surgery	
Bone Wax	
Haemostatic Sponges	Gelfoam/Surgifoam
Oxidized Cellulose Based	Surgicel (Johnson & Johnson), Curacel (Curaspon)
Collagen Based	Instat (Johnson & Johnson), Lyostypt (B-Braun), Hemocol (Pilling-Weck)
Fibrin Sealant Using Wound Fibrinogen	FloSeal (Baxter, formerly Proceed, by Centerpulse)
Bi-Component Fibrin Sealant	Tissucol/Tisseel (Baxter), Beriplast (Behring), Hemaseel (Haemacure), CoStasis (Cohesion Tech)

OC was first introduced by Frantz in 1942 [4]. It is produced by regenerating cellulose after the decomposition of wood pulp. It is available as loosely knit fibrillar material and can be easily packed in cavities (usually along with a hemostatic sponge) to achieve hemostasis. It functions by decreasing the pH of its surrounding tissues. This results in red cell lysis and the formation of acid haematin, causing brownish discolouration. The acidic environment acts as a caustic agent and helps in generating an artificial clot [1]. OC also causes the mechanical tamponade effect and ceases blood loss [5].The coagulum expands in volume secondary to fluid encapsulation. The additional advantage of lowering the pH lies in its antimicrobial effect [2]. The acidic nature also increases the inflammation of the surrounding tissue and has been reported to result in delayed wound healing [5]. Depending on the amount of OC used, the dissolution time may vary from two to six weeks [4].

Surgicel has been reported to cause cord compression after thoracotomy by Brodbelt et al. [6]. In their report using three cases, Surgicel had passed through the intervertebral foramen and resulted in spinal cord compression. They recommended that the smallest amount of Surgicel should be used and it should be removed as completely as possible after its purpose is achieved.

Cases of volume compression from OC have been reported to result in blindness, compressive optic neuropathy, and compression of the optic chiasma [7]. In spine-related literature, we found only two recent reports of the cauda equine syndrome following lumbar microdiscectomy after retained hemostatic agents [8-9]. In both these cases, the hemostatic agent (Surgicel) was left behind around the annular defect. In our case, the reason for leaving OC behind was bleeding from the lytic vertebral body defect.

Though it is recommended to remove OC after hemostasis, in clinical practice, however, Surgicel is often left behind in surgical cavities in the form of hemostatic plugs with gelatine foam [1].

In our case, there was massive bleeding from the lytic vertebral body defect. We attempted to remove the OC but the reoccurrence of bleeding led us to pack the cavity due to concerns of postoperative hematoma development. 

In retrospect, we may have waited longer to remove the OC during the index procedure. It is important to be aware that OC is likely to experience growth postoperatively that could lead to neural compression. We suggest that a complete armamentarium of hemostatic agents should be available in cases where significant blood loss is expected.

## Conclusions

Hemostatic agents are routinely used in spine surgery. OC (Surgicel) expands in volume, so care should be taken before packing it in closed cavities and all attempts should be made to remove any excess Surgicel.
